# The Prenatal and Early Life Exposome: Shaping Health Across the Lifespan∗

**DOI:** 10.1016/j.jacadv.2023.100807

**Published:** 2024-01-05

**Authors:** Omar Hahad, Sadeer Al-Kindi

**Affiliations:** a Department of Cardiology–Cardiology I, University Medical Center of the Johannes Gutenberg University Mainz, Mainz, Germany; b German Centre for Cardiovascular Research (DZHK), Partner site Rhine-Main, Mainz, Germany; c Cardiovascular Prevention & Wellness Center for CV Computational & Precision Health, Houston Methodist DeBakey Heart & Vascular Center, Houston, Texas, USA

**Keywords:** blood pressure trajectories, exposome, prenatal urban exposures

**Figure:**
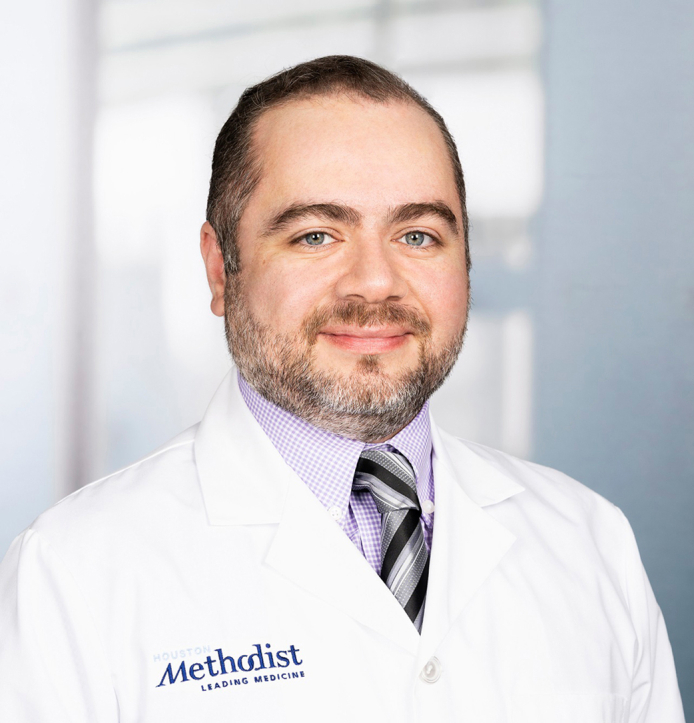

The exposome framework takes a panoramic, all-encompassing view of the environmental influences shaping health over a lifespan. It moves beyond examining exposures in isolation to appraise the totality of external and internal exposures as an interconnected dynamic system that interacts to guide trajectories of health or disease.^1^ This more holistic conceptualization encompasses the whole matrix of chemical, biological, physical, social and psychosocial exposures—from lifestyle behaviors to ambient pollution; nutritional diet to gut microbiome; built geography to natural spaces.^1^ Consideration of the full set of environmental interactions over a life course allows more granular elucidation of the multidimensional drivers underpinning population health distributions. Appreciating this complex interplay of exposures through an exposome lens offers new opportunities to elucidate subtly interconnected risks and informs personalized intervention strategies to optimize healthy trajectories from conception onward.^1^^,^^2^

Prenatal and early life represent sensitive developmental windows where exposures can exert lasting impacts on health trajectories. The developmental origins of health and disease paradigm emphasizes how early environmental exposures may permanently alter structure and function of organs/tissues, with lifelong ramifications.^3^ These formative stages harbor special susceptibility, as exposures can disrupt crucial development and trigger epigenetic shifts in gene regulation that precipitate later-life chronic illness. Appreciating the cumulative, compounding nature of lifetime exposures, the influences of gestational and infant environments set trajectories of resilience or risk. Characterizing those early potentially modifiable factors offers opportunities for primordial prevention efforts to promote public health. Elucidating exposome profiles during early development is imperative to inform clinical and population prevention strategies that can optimize lifelong well-being by safeguarding the earliest building blocks of health.

A step forward is the study in this issue of the *JACC: Advances* by Soares et al^4^ examining the links between prenatal urban environmental exposures and the trajectories of blood pressure from childhood to early adulthood. Employing a comprehensive exposome-wide association study approach, data on systolic blood pressure (SBP) and diastolic blood pressure (DBP) were collected from a large UK birth cohort comprising 7,454 participants. The 43 prenatal urban exposures encompassed factors such as noise, air pollution, built environment, natural spaces, traffic, meteorology, and food environment. Using linear spline mixed-effects models, the study assessed the associations of each exposure (39 in total after removing 1 of highly correlated pairs with blood pressure trajectories). Rigorous replication efforts were undertaken across 4 independent European cohorts, totaling up to 9,261 participants. The findings from the discovery analyses revealed higher humidity to be associated with a swifter increase in childhood SBP, while elevated temperature was linked to a slower rise in SBP during this developmental stage after accounting for multiple testing. Moreover, heightened humidity and air pollution levels were associated with accelerated DBP increase in childhood, coupled with a decelerated rise in adolescence. Only minor evidence of sex differences in these associations was observed. Notably, there was limited evidence of associations between other exposures and changes in SBP or DBP. The replicated results across different cohorts substantiate the notion that heightened prenatal humidity and temperature could indeed modulate blood pressure alterations throughout childhood (Figure 1).

**Figure 1:**
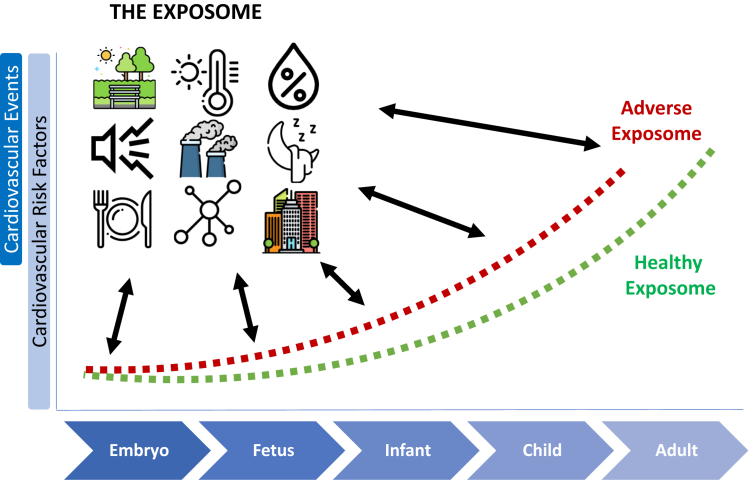
**The Exposome Contributes to Shaping Health Throughout the Life Course** Key environmental factors at different life stages including exposures during fetal development, infancy, childhood, adolescence, early adulthood, and beyond contribute to the shaping of an individual's overall health throughout the life course. Environmental exposures can influence cardiovascular health in a positive or negative direction.

There is evidence suggesting a potential modulation of blood pressure trajectories in childhood/offspring by prenatal outdoor temperature and humidity.^5^, ^6^, ^7^ In general, these studies have indicated that variations in prenatal environmental conditions may exert influences on cardiovascular development in utero, subsequently impacting blood pressure patterns later in life. However, the absence of distinct associations between other well-established environmental-related drivers of blood pressure, such as noise and air pollution,^8^, ^9^, ^10^ in this study is somewhat an unexpected finding and warrants further exploration. The absence of such associations in the present study may be attributable to various methodological considerations, encompassing issues related to the assignment and reliability of exposure assessment methods resulting in exposure misclassification, as well as a spectrum of statistical challenges such as potentially low variance in the exposure distribution. In the context of numerous exposures and outcomes, statistical approaches must deal with the potential for differential power in detecting associations, influenced by the precision of exposure estimation. The conventional approach of favoring the best-estimated exposure in analyses may oversimplify the reality of health and disease often arising from a combination of exposures. Herein, a further limitation of the present statistical approach includes the absence of consideration for potential interactions or nonlinear associations. Data science approaches may help to facilitate processes such as feature selection, dimensionality reduction, clustering to discern patterns, and categorize correlated variables, identifying nonlinear relationships and interactions among exposures, thereby contributing to a nuanced understanding.^1^ Lastly, it is also important to note that a single point estimate of exposures may inadequately capture nuanced fluctuations in blood pressure over time, given the substantial temporal and spatial variability in environmental exposures.

Moving forward, intervention trials present a logical next step to establish causality and advance our capability to harness the exposome for optimizing health. Specifically, experimental studies modifying components of the prenatal exposome hold immense potential to elucidate causal associations with offspring outcomes. For instance, investigators could manipulate exposure to specific climate variables, pollution levels, noise, built environment factors, or other exposures. Comparing measurable outcomes in exposed vs unexposed groups would clarify causal effects and longitudinal follow-up would illuminate enduring impacts on chronic disease risk trajectories. Ultimately, such intervention trials represent a critical translation of exposome science from observational insights to evidence-based recommendations regarding modifiable targets for disease prevention. Confirming critical elements of the exposome at each stage of life amenable to intervention, and quantifying resultant health improvements, will stimulate action from both a clinical and public health perspective. Aligning rigorous experimental exposome research with implementation science approaches will expedite our capability to craft healthier environments supporting optimal well-being.

In conclusion, this impressive study sheds light on the nuanced associations between a range of prenatal urban environmental exposures and blood pressure trajectories, emphasizing the potential influence of humidity and temperature during the early stages of life. Further high-quality studies are warranted to examine the association between prenatal exposomic fingerprints and health outcomes throughout childhood and later life.

## Funding support and author disclosures

Dr Al-Kindi is funded by the 10.13039/100009720Jerold B Katz Foundation. Dr Hahad has reported that he has no relationships relevant to the contents of this paper to disclose.
